# Analysis of morphological and molecular composition changes in allergenic *Artemisia vulgaris* L. pollen under traffic pollution using SEM and FTIR spectroscopy

**DOI:** 10.1007/s11356-016-7554-8

**Published:** 2016-09-07

**Authors:** J Depciuch, I Kasprzyk, E Roga, M Parlinska-Wojtan

**Affiliations:** 1 Institute of Nuclear Physics Polish Academy of Sciences, 31342 Krakow, Poland; 2 Department of Environmental Biology, Faculty of Biology and Agriculture, University of Rzeszow, Zelwerowicza 4, 35-601 Rzeszow, Poland

**Keywords:** FTIR spectroscopy, *Artemisia*, Allergenic pollen, Protein secondary structure, Traffic pollutants, Biomonitoring

## Abstract

Nowadays, pollen allergy becomes an increasing problem for human population. Common mugwort (*Artemisia vulgaris* L.) is one of the major allergenic plants in Europe. In this study, the influence of air pollution caused by traffic on the structure and chemical composition of common mugwort pollen was investigated. Scanning electron microscopy (SEM), Fourier transform infrared spectroscopy (FTIR), and curve-fitting analysis of amide I profile was applied to assess the morphological and structural changes of mugwort pollen grains collected from sites with different vehicle pollution levels. Microscopic observations support the conclusion, that the higher the car traffic, the smaller the pollen grains. The obtained results clearly show that air pollution had an impact on different maximum absorbance values of individual functional groups composing the chemical structure of pollen. Moreover, air pollution induced structural changes in macromolecules of mugwort pollen. In pollen collected from the unpolluted site, the content of sporopollenin (850 cm^−1^) was the highest, whereas polysaccharide concentration (1032 cm^−1^) was the lowest. Significant differences were observed in lipids. Pollen collected from the site with heavy traffic had the lowest content of lipids at 1709, 2071, and 2930 cm^−1^. The largest differences were observed in the spectra regions corresponding to proteins. In pollen collected from unpolluted site, the highest level of β-sheet (1600 cm^−1^) and α-helix (1650 cm^−1^) was detected. The structural changes in proteins, observed in the second derivative of the FTIR spectrum and in the curve-fitting analysis of amide I profile, could be caused inter alia by air pollutants. Alterations in protein structure and in their content in the pollen may increase the sensitization and subsequent risk of allergy in predisposed people. The obtained results suggest that the changes in chemical composition of pollen may be a good indicator of air quality and that FTIR may be successfully applied in biomonitoring.

## Introduction

Plant pollen is an important source of airborne allergens. It causes allergic rhinitis, conjunctivitis, and asthma. Nowadays, a significant increase of inhalant allergy incidence is observed, therefore it can be considered as an epidemic (Chu et al. 2014; Sozańska et al. 2014). It is estimated that 30–40 % of human population is sensitized to pollen (D’Amato et al. 2007; Singh and Mathur 2012). This phenomenon escalates in polluted and urbanized areas (Sozańska et al. 2014; Timm et al. 2016). *Artemisia vulgaris* L. (common mugwort) is one of the major herbaceous allergenic plants in Europe. The occurrence of allergy against mugwort has been estimated between 11.4 and 13.8 %, with maximum of 45 % in Hungary (Stach et al. 2007; Burbach et al. 2009).

Currently, one of the main sources of air pollution in cities are cars (Rebolj and Sturm 1999). Dominant pollutants are nitrogen, sulfur and carbon oxides, ozone, and PM10 (Wang et al. 2008). Many authors claimed that air pollution affects the decrease in pollen viability and production; pollen grains may be smaller, often deformed or crashed (Majd et al. 2004; Rezanejad 2009; Lu et al. 2014; Sénéchal et al. 2015; Kaur et al. 2015). Chemical pollution may influence the biochemical composition of pollen. Rezanejad (2009) observed an increase of phenols and flavonoids contents. Conversely, Majd et al. (2004) detected a decrease of protein content in pollen. Consequently, chemical air pollutants may alter the allergenic potency of pollen by inducing substantial molecular changes of proteins, consisting in their nitration and oxidation (Alscher et al. 1997; Bryce et al. 2010; Karle et al. 2012; Ackaert et al. 2014; Lu et al. 2014). Guedes et al. (2009) stated differences in protein profiles in pollen sampled from polluted sites. Rogerieux et al. (2007) showed a decrease of the *Phleum pratense* allergen detected by IgEs. Chemical pollutants favor the production of pathogenesis related proteins (PR), which are also allergens, that partially explains the intensification of pollen allergy occurrence in urbanized and industrial areas (Sinha et al. 2014). All these facts may have serious clinical implications for allergic and inflammatory diseases and make proper prophylaxis, diagnosis, and treatment difficult (Majd et al. 2004; Ghiani et al. 2012; Karle et al. 2012; Todea et al. 2013).

Traditional aerobiological monitoring has limited possibilities to detect the negative impact of abiotic stress on pollen. Only morphological changes of pollen grains using optical or scanning electron microscopes can be observed, simultaneously measuring their size and estimating their viability (Kaur et al. 2015). Mid Infrared Fourier Transform spectroscopy gives more possibilities, as it provides information about the chemical composition, as well as about qualitative and quantitative changes in the investigated samples. In FTIR technique, in order to observe a peak from a compound, the value of its dipole moment has to change. These very quick changes of the dipole moment occur under IR radiation causing the formation of very large peaks in the FTIR spectrum, allowing to measure the functional bonds of compounds (Larkin 2011). High resolution, fast measurements, low cost, and minimal material preparation are some of the many advantages of this method (Pappas et al. 2003; Zimmermann 2010; Zimmermann and Kohler 2014; Zimmermann et al. 2016). For these reasons, FTIR spectroscopy is being increasingly used in the fields of agriculture, biology, ecology, biochemistry, and medicine (Movasaghia et al. 2008; Rubio-Diaz et al. 2010; Singh et al. 2012; Jiang et al. 2015; Depciuch et al. 2016a, b). This method was also applied in palynology as an effective tool in phylogenetic taxonomy (Pappas et al. 2003, Gottardini et al. 2007; Zimmermann 2010; Zimmermann and Kohler, 2014; Bağcıoğlu et al. 2015). Intensive studies on pollen biochemical compounds allowed creating a spectra library for many species, which enables the identification of pollen (Gottardini et al. 2007; Dell’Anna et al. 2009; Guedes et al. 2014; Zimmermann 2010; Zimmermann et al. 2015, 2016). Buta et al. (2015) used FTIR to determine the links between the chemical structure, viability and germination of pollen of *Saintpaulia* genotypes. Zimermann and Kohler (2014) showed distinct inter-annual variations of biochemical constituents of Pinaceae specimens, which were explained by different course of weather in the period of pollen differentiation. Until now, FTIR was not applied to detect differences in pollen spectra of individuals of the same species induced by air pollution. The possibility to quickly determine the relation between pollution and molecular composition of allergenic pollen is of particular importance for sensitized people.

The reaction of plants to environmental pollution depends on the type of pollutants, but their concurrent influence is more damaging than the influence of an individual pollutant (Adaros et al. 1991). In this study, the assumption that car originated pollution strongly affects the morphology and molecular composition of allergenic common mugwort pollen grains is experimentally verified. Infrared spectroscopy (FTIR) and curve-fitting analysis of amide I profile were applied to assess the quantitative and structural changes in the macromolecule’s compound of common mugwort pollen collected from sites with different vehicle pollution levels. The important question was, whether the chemical composition of pollen is a good indicator of air quality, and if FTIR may be applied in biomonitoring.

## Materials and methods

### Pollen sampling and measurements

**Fig. 1:**
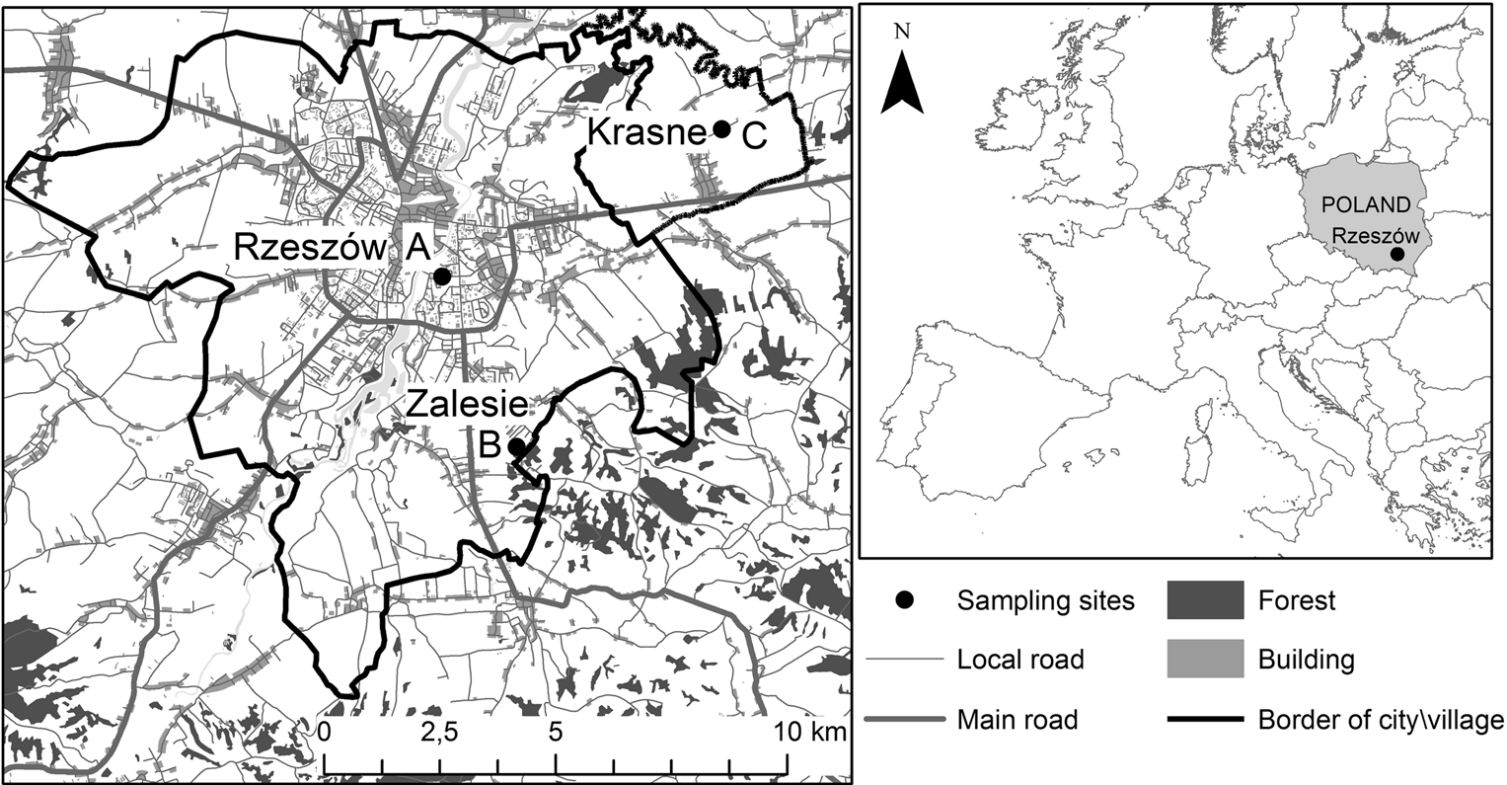
Location of sampling sites

This study was carried out in the first half of August 2015 in Rzeszow, SE Poland. The pollen of common mugwort was collected at three sites with different car traffic intensity: Rzeszow (site A, big crossroad in the city center), Zalesie (site B, area without traffic), and Krasne (site C, moderate traffic), Table 1. The sites A and C were located at a distance of 7 km and sites B and C 9 km in a straight line from each other (Fig. 1). All sites were classified as rural habitats with urban soil type. They were similar in terms of sun exposition, land relief, slope, a.s.l. Thus, the car-related pollution was the most important parameter differentiating these locations. Also, a difference in the mean temperature values was observed, with the highest value in city center, site A. In all studied areas, the concentrations of air pollutants did not exceed the critical values; however, in the center of Rzeszow, they were higher than outside the city (Table 1). It is known that there are substantial differences in air pollutant concentrations in the vertical profile, with the highest values near ground level (Wang et al. 2008). Therefore, site A was regarded as the most polluted location.

**Table 1:** Air quality classes (A–B) and mean concentrations of chosen air pollutants in respect to the critical values (according to EU Directive adapted to national low, 2008/50/WE)

Sites	SO_2_ 20 (μg/m^3^) Annual average	NO_2_ 30 (μg/m^3^) Annual average	CO 10000 (μg/m^3^) 8-h average	O_3_ Days above threshold-25	Pb in air 0.5 (μg/m^3^) Annual average	PM10 40 (μg/m^3^) Annual average	PM2.5 25 (μg/m^3^) Annual average
Rzeszow—A	A; 5–6	A; 17–25	A; 3501–4858	A; 8–9	A; 0.0095–0.02	A; 21–30	B; 21–32
Zalesie—B	A; 3–4	A; 5–8	A; 1501–2500	A; 8–9	A; 0.0055–0.009	A; 11–20	A; 11–15
Krasne—C	A; 3–4	A; 9–10	A; 1501–2500	A; 10–12	A; 0.0055–0.009	A; 11–20	A; 16–20

The results were obtained from http://www.wios.rzeszow.pl/wp-content/uploads/2015/05/ocena_jakosci_pow_2015.pdf

Class A indicates concentrations below the critical value. Class B indicates concentrations above the critical value

*PM* particular matters

The pollen was sampled directly from inflorescences during 1 week of full flowering period, from five randomly chosen plants from each site. In total, pollen was collected from 15 plants. Each plant had the same height and was well branched with many inflorescences. The pollen was dried and kept in the same conditions. From each plant, about 1 g of pollen was collected. This amount was sufficient to conduct scanning electron microscopy (SEM) and spectroscopic analysis. Subsequently, a part of the pollen was prepared for microscopy analysis. From each site, minimum of 100 pollen grains were imaged by microscopy. Their polar axis was measured at 400× magnification under a light microscope using NIS software. Their equatorial axis was imaged using the SEM at 8000× magnification using Vega TC software. These two types of microscopes were used, because on microscopic glass slides, the pollen grains were almost always in a polar position, whereas in SEM images, they were mostly in equatorial position.

### SEM imaging

Scanning electron microscopy was carried out on a TESCAN VEGA 3 SBH instrument equipped with a tungsten cathode. Directly after being dried, pollen was deposited a SEM stub sample holder covered with a carbon patch. Uncoated samples were imaged in high vacuum mode at 1 kV accelerating voltage using the SE detector.

### FTIR measurements

FTIR spectroscopy measurements were performed using a Vertex 70 (Bruker) spectrometer applying the attenuated total reflectance (ATR) technique. Multiple internal reflections ATR with germanium crystal was used. Two milligrams of pollen was used for spectral analysis. Spectra were collected in the 4000–400 cm^−1^ range by co-adding 64 scans at 4 cm^−1^ resolution. To obtain the absorbance spectrum of pollen, the ATR spectra were corrected for wavelengths depending on the penetration depth of the infrared beam into the sample. For each sample, three spectra were acquired and averaged. This procedure was performed for each pollen sample separately (15 samples). Moreover, the ATR technique allowed measuring only the surface of the samples. In order to determine the structural changes, the second derivatives of the individual spectra were calculated.

### Data analysis

The analysis of the secondary structure of the proteins was carried out by curve fitting for the amide I bond in the 1700–1610 cm^−1^ range, using the GRAMS AI software from Thermo Scientific. Unfortunately, the range of the amide I overlaps with the range of the water spectrum. Therefore, in this range, the signal from the water background was often observed. Complete drying of pollen, which was necessary to perform SEM imaging, ensured that the resulting FTIR spectra in the range between 1600 and 1700 cm^−1^ do not contain water background signals. The second derivatives were calculated from the ATR–FTIR spectra after smoothing over two consecutive points. The absorption bonds at low wavenumbers were free of features from water vapor as judged from the peaks above 1750 cm^−1^. A straight baseline passing through the ordinate at 1700 and 1610 cm^−1^ was subtracted before the curve fitting. The baseline was again modified by the least-squares curve-fitting software, which allows for a horizontal baseline to be adjusted as an additional parameter to obtain the best fit. The second derivative spectrum was used to determine the initial peak positions for curve fitting, and the peaks were fitted using Gauss functions. The area under the entire absorption bond was considered as 100 %, and each component after fitting was expressed as a percent fraction. Each spectrum was normalized automatically using OPUS software with the normalization option.

### Statistical analysis

The Shapiro-Wilk and Brown-Forsyth tests were used to check the normality and homoscedasticity, respectively. One-way ANOVA was applied to detect any differences in the mean values of maximum absorbance for each individual wavenumber corresponding to the main absorption bands. Then, the Tukey test for multiple pairwise comparisons of means between sites was used. The comparison of the size of pollen grains was done by the Kruskal-Wallis procedure and Dunn post hoc test, because the variances were not homogeneous. The statistical hypotheses were tested with *α* ≤ 0.05. In order to indicate the groups of the individual plants with the highest similarity in respect to the entire absorbance spectra, hierarchical clustering analysis (HCA) with Euclidean distance and Ward’s algorithms was applied. Statistical and multidimensional analysis was done using STATISTICA ver. 9 and PAST software.

## Results

### Shape and size of pollen grains

**Fig. 2:**
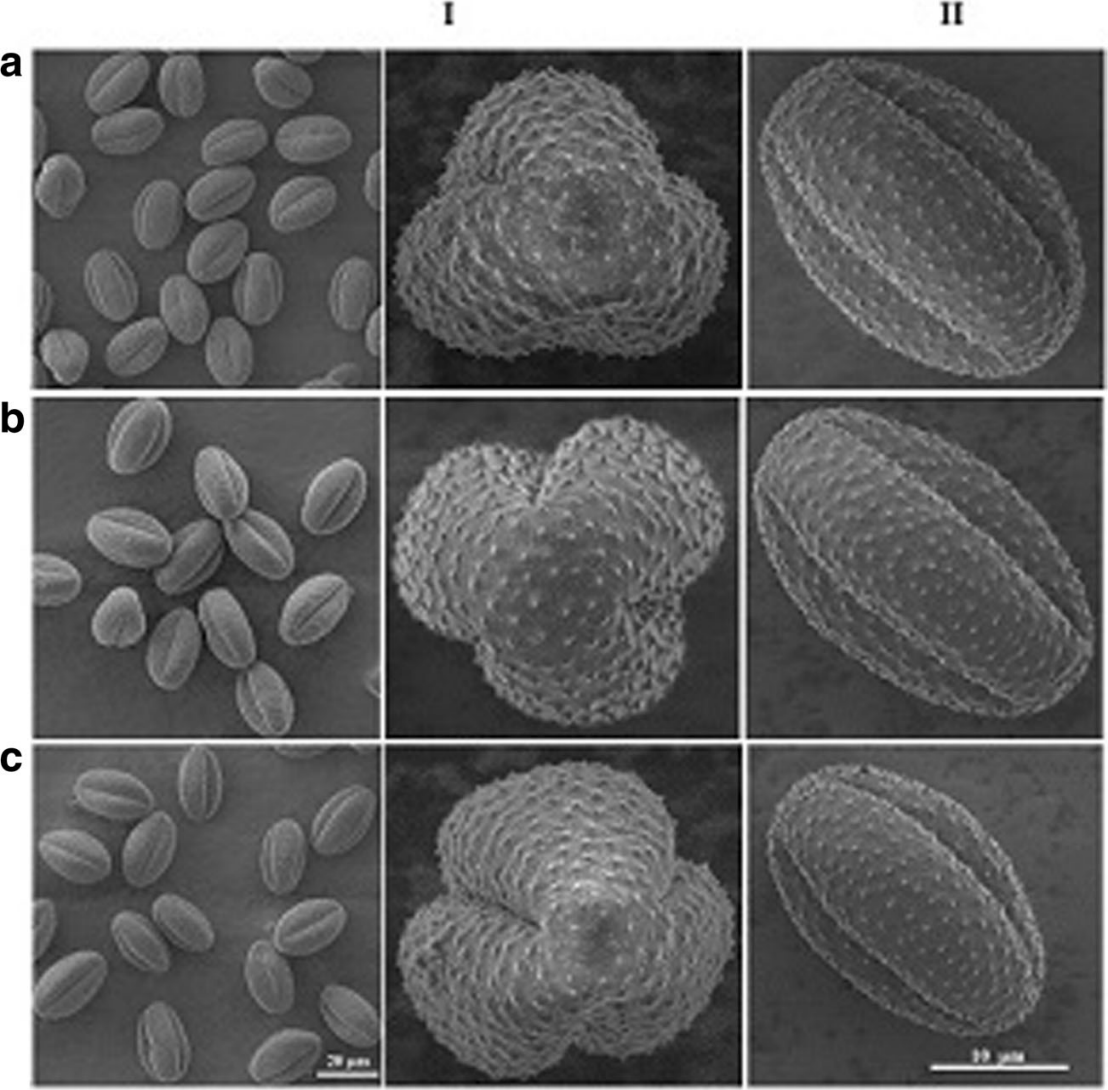
SEM images of *A. vulgaris* pollen grains (*I* polar view, *II* equatorial view) collected from *A* Rzeszow, *B* Zalesie, and *C* Krasne. The *scale bars* are the same for all overview images

Figure 2 shows a set of SEM images of common mugwort pollen grains collected from sites A, B, and C. The SEM imaging did not reveal any differences in shape and surface of the pollen grains collected from the three regions as well as any physical degradation. Pollen grains were well developed, not broken, without any contaminants on their surface visible under the light or electron microscopes. No distinct differences in the colpi and exine features could be noticed (Fig. 2).

The only difference was the size of the pollen grains. The ones collected from sites with traffic, A and C, had similar polar axis sizes (20.623 and 20.518 μm, respectively) and were significantly smaller than those from site B by an average of 1.372 μm (Kruskal-Wallis test *H* = 46.36; *p* = 0.000; Table 2). The longest equatorial axis was characteristic for pollen grains collected from site B with no traffic, which is visible in Fig. 2. Their equatorial axis was longer by an average of 3.504 μm from those collected at the site A and by 1.876 μm from those collected at the site C (Kruskal-Wallis test *H* = 146.28; *p* = 0.000; Table 2).

**Table 2:** Results of descriptive statistics of polar and equatorial axis of pollen grains collected in three sites (Rzeszow—A, Zalesie—B, Krasne—C)

	Polar axis					Equatorial axis				
Site	Mean (μm)	Min (μm)	Max (μm)	SD (μm)	CV%	Mean (μm)	Min (μm)	Max (μm)	SD (μm)	CV%
Rzeszow—A	20.623^a^	14.61	24.38	1.901	9.2	22.114^a^	19.09	25.13	1.194	5.4
Zalesie—B	21.943^b^	16.21	26.3	1.724	7.9	25.618^b^	21.89	29.60	1.701	6.6
Krasne—C	20.518^a^	16.15	27.29	2.138	10.4	23.742^c^	19.35	27.26	1.654	6.9

Arabic letters (a, b, c) indicate homogeneous groups in respect to the pollen grains’ size. The groups were distinguished on the base of nonparametric Kruskal-Wallis test and Dunn test for multiply comparisons. The differences were statistically significant with *p* < 0.0000

*SD* standard deviation, *CV*% the variability coefficient

### Molecular composition

**Fig. 3:**
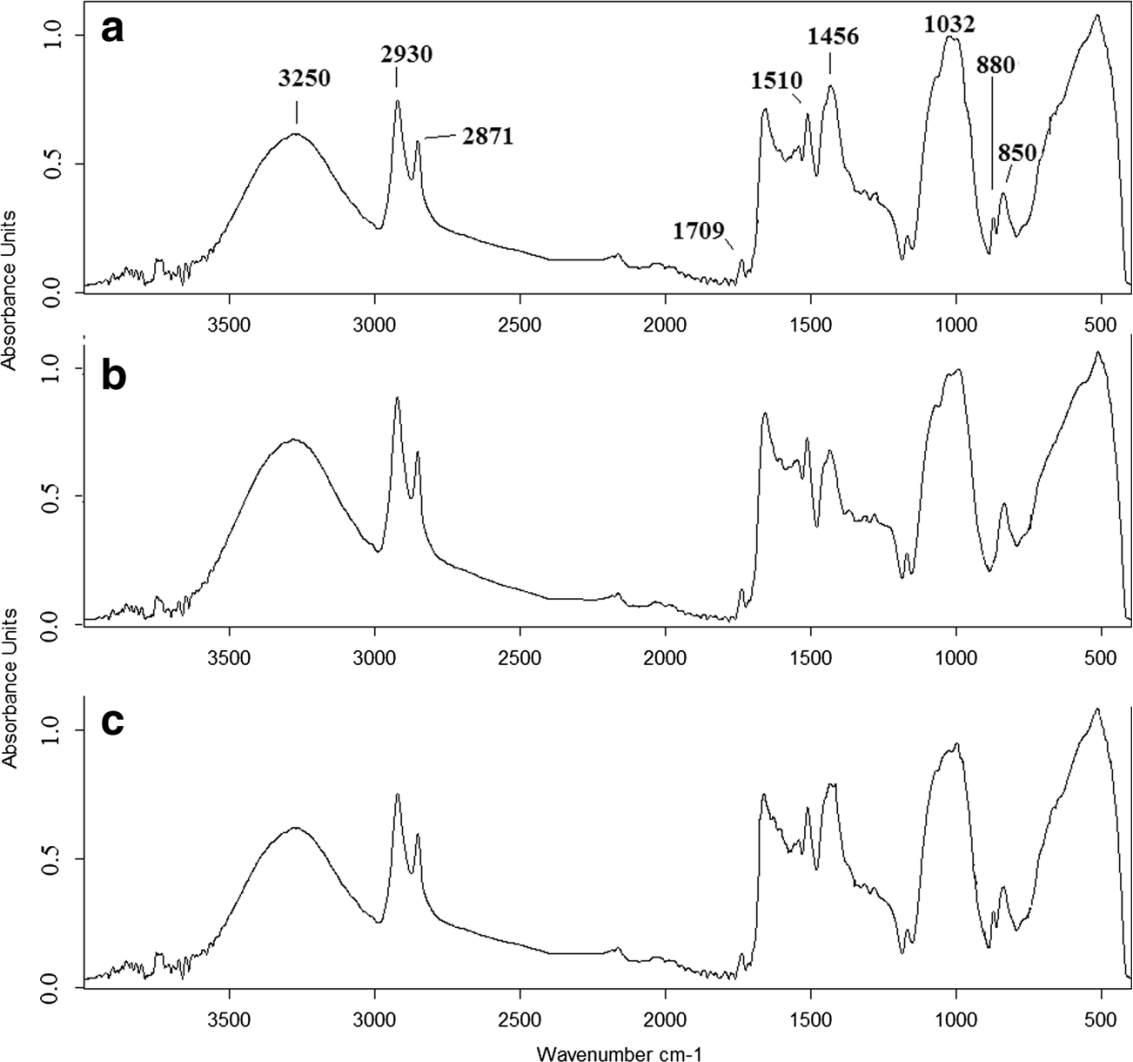
Offset of average FTIR spectra of common mugwort pollen from *A* Rzeszow, *B* Zalesie, and *C* Krasne. The peaks, which showed the most apparent differences, are *encircled*

No changes in pollen morphology and exine structure could be observed by SEM. Therefore, chemical analysis was performed by FTIR spectroscopy to detect the possible changes in pollen biochemical composition. Figure 3 shows the offset of the FTIR spectra indicating specific bonds for pollen from each site. Main absorption bonds and corresponding assignments are presented in Table 3. For each pollen sample, the same absorption bonds corresponding to nucleic acids, proteins, polysaccharides, lipids, and water were identified (Table 3, Fig. 3). The low wavenumber region of the FTIR spectrum originates from the chemical bonds of aromatic ring vibrations of sporopollenins (850 and 880 cm^−1^), polysaccharides (1032 cm^−1^), and proteins (1456, 1510 cm^−1^, respectively). The peaks at 1709 cm^−1^ correspond to stretching vibrations of the C=O group, which indicates the presence of lipids. Vibrations observed in the FTIR spectrum at 2871 and 2930 cm^−1^ originate from the C-H vibrations of alkyl group and -CH_2_ and -CH_3_ vibrations of lipids, proteins, and carbohydrates. The peak at 3250 cm^−1^ corresponds to the water remaining in pollen (Rubio-Diaz et al. 2010; Mohani et al. 2014; Rather et al. 2014).

**Table 3:** Values of wavenumbers with the corresponding vibrations (Rubio-Diaz et al. 2010; Mohani et al. 2014; Rather et al. 2014)

	Wavenumber (cm^−1^)	FTIR
1	850	C=C trans double bonds from sporopollenin
2	880	C=C trans double bonds from sporopollenin
3	1032	Stretching vibrations of C-O-C group–polysaccharides
4	1456	Bendig vibrations of C-N-H (amid II)–protein
5	1510	Bendig vibrations of C-N-H (amid II)–protein
6	1709	Stretching vibrations of C = O group–lipids
7	2871	C-H vibrations of alkyl group and -CH_2_ and -CH_3_ vibrations of lipids, proteins, and carbohydrates
8	2930	C-H vibrations of alkyl group and -CH_2_ and -CH_3_ vibrations of lipids, proteins, and carbohydrates
9	3250	Deformation vibrations of O-H–water

The FTIR spectra (Fig. 3) show that depending on the location of the material sampling, the chemical composition of pollen was different. In fact, although the measured samples were pollen of the same plant species, no two identical FTIR spectra were obtained. The highest level of aromatic ring vibrations of sporopollenin at wavenumber 850 cm^−1^was noticed in pollen collected from the site B. Interestingly, in the same spectrum, aromatic ring vibrations of sporopollenin at wavenumber 880 cm^−1^ corresponding to the C=C trans double bonds were not present (Fig. 3). These vibrations were observed in pollen collected from sites located close to the streets (A and C). It was found that pollen from site A had significantly more polysaccharides (1032 cm^−1^) than the samples from sites B and C, respectively (Anova *F* = 1368.38; *p* = 0.000). The samples differed also with respect to the protein content. Pollen collected from site B exhibited the highest value of maximum absorbance corresponding to proteins at 1510 cm^−1^ (Anova *F* = 9208.48; *p* = 0.000). In pollen collected from site A, the significantly highest level of proteins at 1456 cm^−1^ compared to sites B and C was observed (Anova *F* = 470.61; *p* = 0.000). Pollen collected from sites A and B differed significantly with respect to the lipids content. In pollen from site A, the value of the maximum absorbance at 1709 cm^−1^ was the lowest (Anova *F* = 336.81; *p* = 0.000). Pollen collected form site B presented the highest lipids content (maximum of absorbance at 2871, 2930 cm^−1^) as well as water (3250 cm^−1^) compared to pollen from sites A and C, respectively (Fig. 3).

**Fig. 4:**
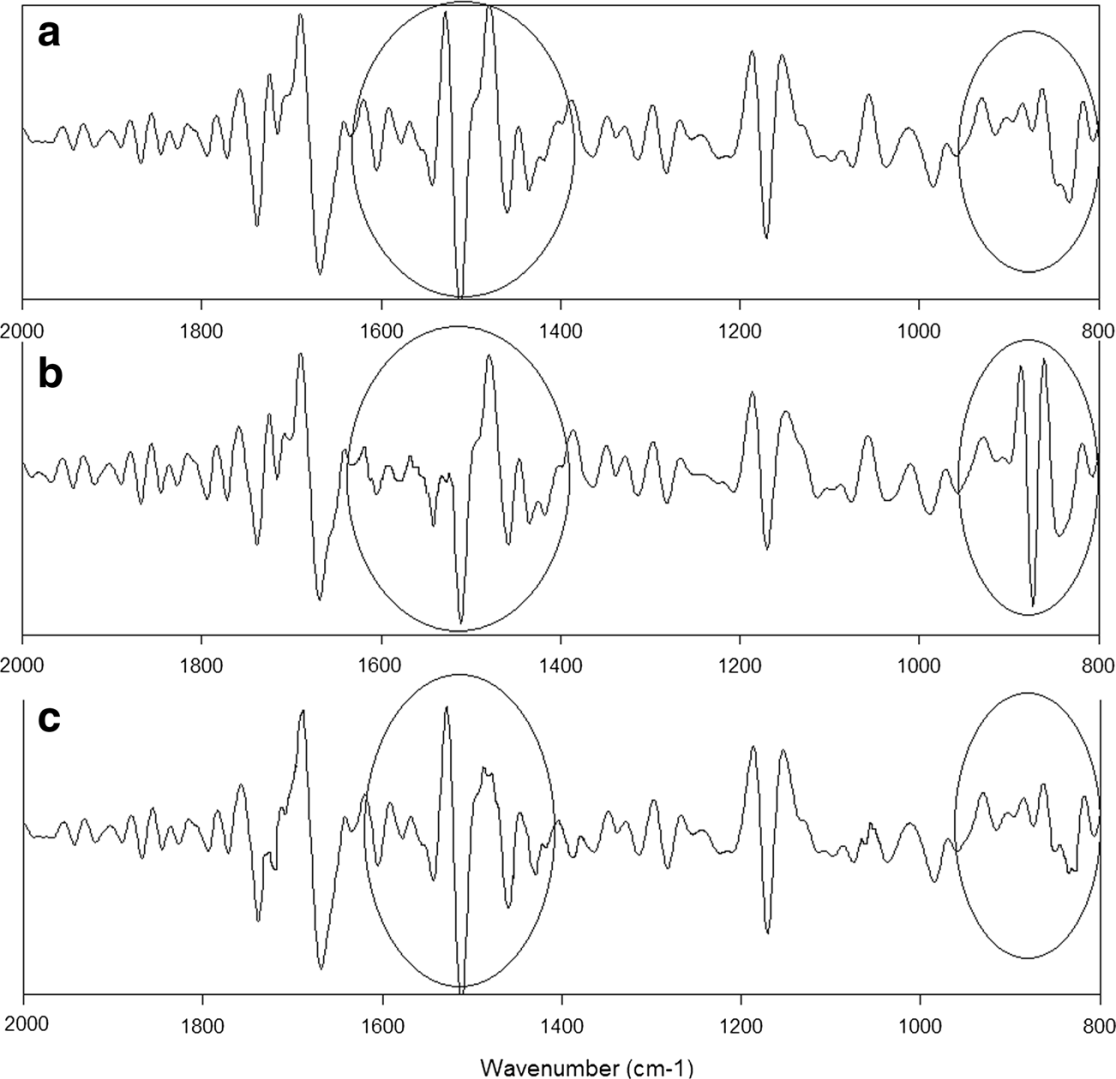
Second derivative of the FTIR spectra of common mugwort pollen from *A* Rzeszow, *B* Zalesie, and *C* Krasne. The peaks, which showed the most apparent differences, are *encircled*

Indeed, the intensities of the individual peaks and the character of spectra (shape of lines, peaks) differ among pollen sampled from the three locations (Fig. 3). It suggests that most probably there are structural differences in the chemical compounds of pollen. To obtain the structural information about individual functional groups that build the chemical structures of pollen, the second derivative was calculated. The molecular changes in the proteins could play a very important role in the pathogenicity of allergy; therefore, the detailed comparisons of the spectra regions corresponding to the vibrations of nucleic acids and proteins were performed. These areas are marked with circles in Fig. 4.

**Fig. 5:**
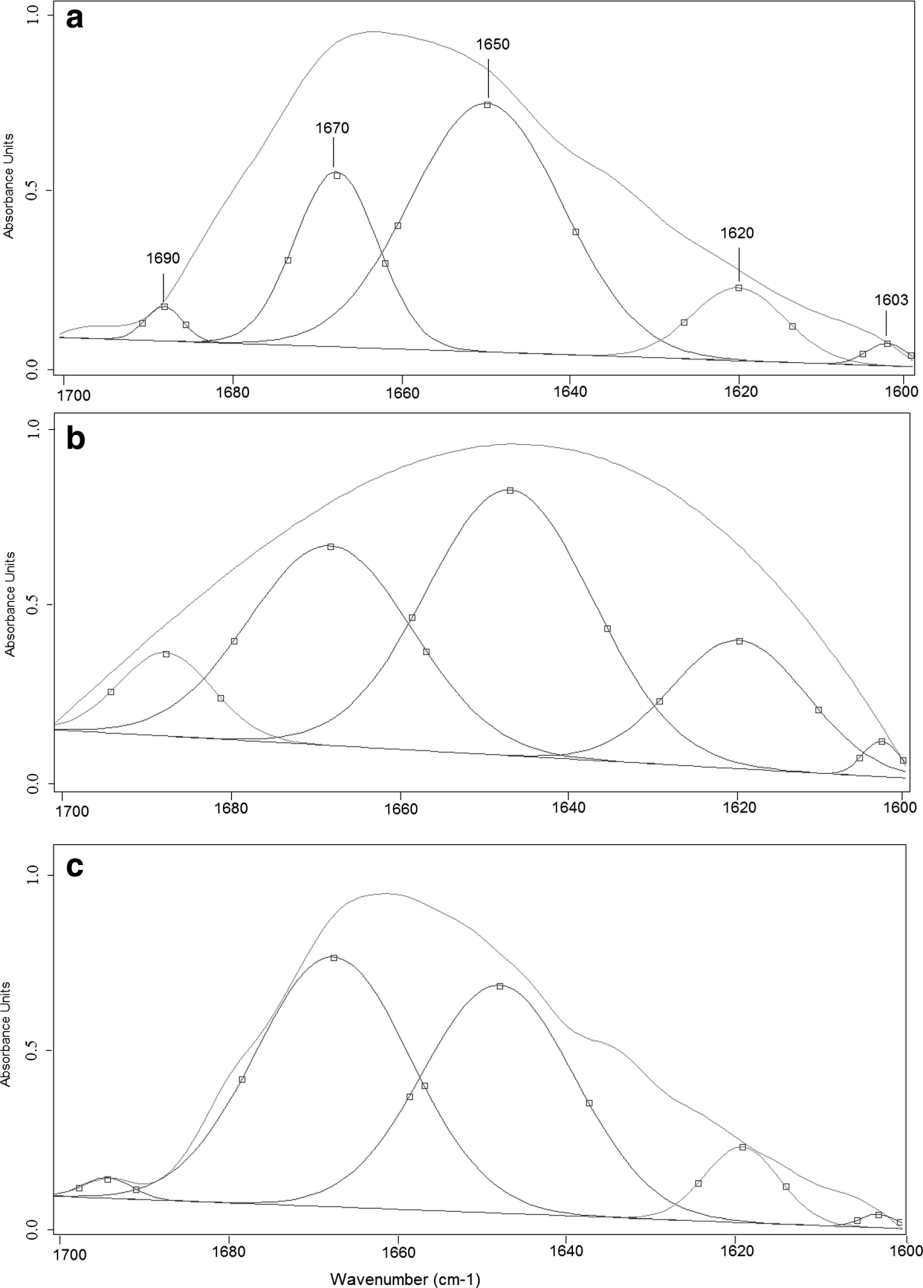
Curve-fitting analysis of the amide I profile of common mugwort pollen from *A* Rzeszow, *B* Zalesie, and *C* Krasne

The second derivatives of the spectra from sites A and C are very similar. Conversely, the character of spectrum of pollen collected from site B is clearly different (Fig. 4). The differences in the second derivative of FTIR spectra from sites A and C in the region corresponding to carotenoids suggests that structural changes in sporopollenin—a biopolymer forming the pollen exine, occur. Moreover, significant variations of the second derivative of the FTIR spectra in the protein region may indicate structural changes in proteins. Thus, curve-fitting analysis of amide I profile was performed (Fig. 5) in order to obtain information about the type of secondary structural changes of α-helix and β-harmonica of proteins. Table 4 shows the percentage content and the vibration type of the protein’s secondary structures for the three analyzed pollen samples.

**Table 4:** Multiply comparisons of the average percentages of the contents of the protein’s secondary structures for each wavenumber (Maury et al. 2005; Mauerer and Lee 2006; Pandey et al. 2010; Misra et al. 2015)

Wavenumber (cm^−1^)	Vibrations	%		
		Rzeszow—A	Zalesie—B	Krasne—C
1600	β-sheet	1.55 a	1.15 b	0.55 c
1620	β-sheet	11.34 a	16.94 b	6.89 c
1650	α-helix	61.34 a	42.37 b	42.12 c
1670	β-turn	23.71 a	31.07 b	48.21 c
1690	β-turn	2.06 a	8.47 b	1.23 c

Arabic letters (a,b,c) indicate homogeneous groups in respect to mean percentage for each wavenumber (vibration) separately based on parametric one-way ANOVA test and Tukey post hoc test for multiply comparisons

As the structural changes of proteins are visible only in amide I bonds (1600–1700 cm^−1^), the curve-fitting analysis was performed only for this region (Fig. 5). All peaks corresponding to the secondary structure of proteins are visible in each of the three pollen spectra. The peaks at 1603 and 1620 cm^−1^ correspond to β-sheet vibrations. The maximum absorbance at wavenumber 1650 cm^−1^ originates from α-helix vibrations. Vibrations observed in the curve-fitting analysis of the amide I profile at 1670, 1690 cm^−1^ correspond to β-turn. The amide I region of FTIR pollen spectrum from site A is very similar to that from site C, but the curve-fitting analysis has revealed more details. The percentage values of the protein’s secondary structures for those two sites are different. Moreover, the amide I region of FTIR pollen spectrum from site B significantly differs from the two others, what was confirmed by ANOVA (Table 4).

Statistically, significant differences in the values of the various proteins structures among sites (ANOVA; for all wavenumbers *p* = 0.0000; Table 4) were observed. In pollen collected from site A, the highest level of β-sheet (1600 cm^−1^) and α-helix (1650 cm^−1^) compared to sites B and C was detected. However, the second peak corresponding to β-sheet (1620 cm^−1^) was the highest in pollen from site B. Moreover, in this pollen sample, the highest level of β-turn (1690 cm^−1^) compared to pollen from sites A and C was detected. The highest level of the other peak corresponding to β-turn (1670 cm^−1^) was noticed in pollen collected from site C.

**Fig. 6:**
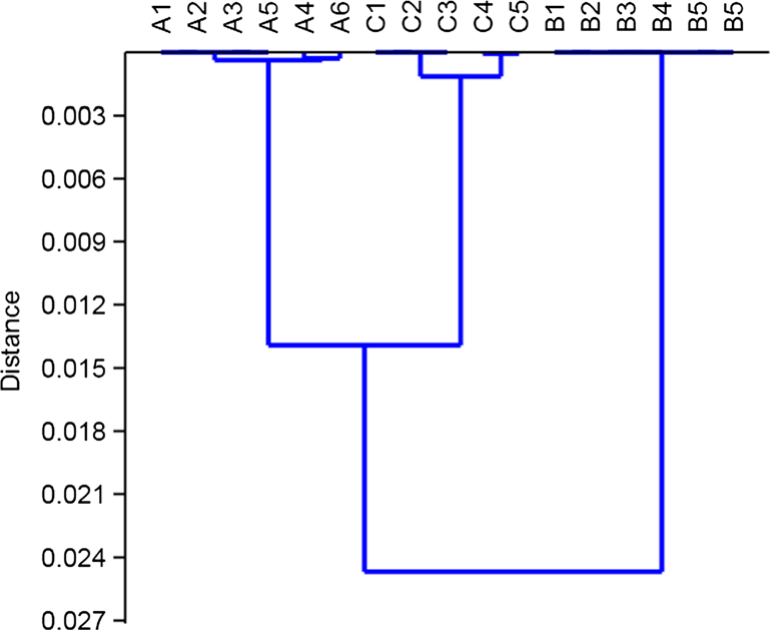
Clustering groups according to the highest similarity of FTIR spectra of pollen from each sites (*A* Rzeszow, *B* Zalesie, *C* Krasne)

The results of HCA indicate clear differences in chemical compounds of pollen collected from the three locations. The analysis of the dendrogram revealed three groups of distinctly different spectra (Fig. 6). Each cluster consists of pollen samples collected from the same site. It should be underlined that the spectra of pollen from site with no car traffic (site B) clearly differed from those with car traffic (sites A and C). The lowest homology of pollen spectra was noticed for pollen sampled from site C. The highest homology was observed in the group collected from site B, for which pollen samples were undistinguishable based on FTIR spectra.

## Discussion

Many authors claimed that plant pollen is a good bioindicator of air quality (Malayeri et al. 2012). They observed that air pollution caused shape changes of pollen grains. The thinner exine may result in higher susceptibility to deformation or fragility (Majd et al. 2004; Rezanejad 2009). In industrial and urbanized areas, pollutants, mainly carbon particles, stick to the pollen grain’s wall (Guedes et al. 2009). This was not observed in our study. Mugwort pollen grains exhibited morphological features typical for this pollen grain type: trizonocolporate, thick exine in the middle of grain thinning toward the furrows. Moreover, the size of pollen grains varied within the standard range of variation given in literature (Piotrowska 2008; Hayat et al. 2010). SEM observations support the conclusion that the higher the car traffic, the smaller the pollen grains. This is in accordance with the findings of Rezanejad (2009). Negative environmental stress strongly influences pollen grains, what is however not always mirrored in their morphology. It affects the pollen viability, germination, and finally reproduction success of the plant (Malayeri et al. 2012; Buta et al. 2015; Lahlali et al. 2014). Kaur et al. (2015) stated that the size and morphology does not depend on traffic, which yet, affects pollen viability. Experimental studies conducted by Kanter et al. (2013) demonstrated that the elevated ozone concentration did not induce ragweed pollen shape and size changes.

Although we did not detect differences in shape of pollen grains or their exine sculpture, we examined the chemical compounds of the samples using infrared (IR) spectroscopy with the ATR technique. Indeed, ATR uses the property of total internal reflection resulting in an evanescent wave. A beam of infrared light passes through the ATR crystal in such a way that it reflects at least once from the internal surface in contact with the sample. This reflection forms the evanescent wave, which extends into the sample. The penetration depth into the sample is typically between 0.5 and 2 μm, with the exact value being determined by the wavelength of light, the angle of incidence, and the indices of refraction for the ATR crystal and the medium being probed. In ATR measurements, the sample thickness does not affect the intensity of the absorbance bonds; in transmission mode, however, very thick samples cause a so-called total absorbance. This can result in similar spectral intensities for samples of different thicknesses. The wavelength dependency of the penetration depth into the sample and the anomalous dispersion of the IR-light result in typical systematic differences between spectra measured using the ATR and the transmission technique (Mirabella 1993). The main advantage of ATR-IR over transmission-IR is the limited path length into the sample. This avoids the problem of strong attenuation of the IR signal in highly absorbing media, such as aqueous solutions. The ATR technique allows for the measurement of the range between 400 and 4000 cm^−1^. This range corresponds to the oscillation frequency of functional groups building organic compounds, e.g., nucleic acid, carbohydrates, proteins, and lipids.

FTIR spectroscopy revealed distinct changes in pollen chemical composition. In the FTIR spectra, differences in the absorbance between pollen collected from the three sites can be observed. The values of maximum absorbance, quantitative and qualitative changes of individual functional groups building the pollen molecules can result from different environmental factors typical for the place where the material was collected (Zimmermann and Kohler 2014). In our experiment, this specific factor was the intensity of the traffic.

Several authors emphasized the negative impact of diesel-exhaust particles on proteins, mainly allergens. Under stress, plants produce more pathogenesis related proteins (PR) and the total protein content increases in pollen (Sinha et al. 2014). Air pollutants directly affect the protein compounds, which build the pollen and can cause mutational changes in the genetic material leading to changes in structure and biochemical properties of the pollen molecules (Majd et al. 2004; Dell’Anna et al. 2009; Ackaert et al. 2014). Elevated ozone concentration causes a decrease of amide I composition (Kanter et al. 2013). This study has shown that the largest differences occur in the protein regions of the spectra. The values of the maximum absorbance differed significantly at each wavenumber. Structural changes in proteins are more visible in the curve-fitting analysis of the amide I region. Moreover, quantification of the α-helices and β-harmonica also differs between the tested samples. Considering the contribution of α-helices and β-sheet in pollen, the question arises, if this situation could be connected only with abiotic stress or the effect of inter-population variability. According to Lahlali et al. (2014), the tolerance to heat may be connected with the α-helices structure. The structural changes of proteins may be also the consequence of mutation in the genetic material caused among others by abiotic factors, e.g., pollutants. Post-translational reversible or irreversible alternations may also occur. Modification in protein content and in their structure may increase the risk of allergy, sensitization in predisposed people as well as immunogenicity of the allergens (Ichinose et al. 2002; Yanagisawa et al. 2006; de Weger et al. 2013; Ghiani et al. 2012; Karle et al. 2012; Ackaert et al. 2014). Hence, quick determination of any changes in chemical compounds in pollen, in particular in the protein group, is of crucial importance. FTIR, in particular, using the ATR technique seems to be an excellent tool for this.

In the FTIR spectra, clear differences in the region of the vibrations of aromatic rings were also detected. The spectrum from site B differed most significantly from the spectra of pollen collected from sites A and C. Spectra from pollen collected from sites A and C contained a peak at 880 cm^−1^ of aromatic ring vibrations of sporopollenins, which built the pollen grain wall, exine. This peak was not observed in the spectrum of pollen from site B; however. a peak from aromatic ring vibrations of sporopollenin at 850 cm^−1^ was noticeable. Fraser et al. (2012) pointed out that sporopollenin is very a recalcitrant biomacromolecule. Our results show that the air pollution could cause the chemical and structural changes in compounds, which build the exine structure.

Heat stress causes a decrease of lipid content, which was detected by Zimmermann and Kohler (2014), as well as by Lahlali‘s team in 2014. The consequence is lower pollen germination potency (Lahlali et al. 2014; Jiang et al. 2015). Pollen lipids play also an important role in the induction and the strengthening of allergy (Risse et al. 2000). They could alter the allergenic properties of proteins and modify the immune response (Bashir et al. 2013). Lipids content and their quantitative changes can be easily detected by infrared spectroscopy (Jiang et al. 2015). Zimmermann and Kohler (2014) found the change in infrared spectra at the peak corresponding to lipids (1745 cm^−1^) in pollen collected from the site with high temperatures. The investigations of Lahlali et al. (2014) observed the reduction of asymmetric and symmetric CH_2_ peaks (peaks at 2922 and 2852 cm^−1^) under heat stress. We also detected changes in the lipid region of the spectrum (peaks at 1709, 2871, 2930 cm^−1^) in pollen collected from the center of Rzeszow city (A), where the mean temperatures in June and July 2015 were about 2 °C higher than in site B. The disturbance in lipid region could also be caused by another stress factor such as air pollution. The research of Pukacki and Chałupka (2003) supports this statement. They observed a modification of lipid content in pine pollen collected from polluted areas. These changes concerned fatty acids and phospholipids, which content was decreased. Also, elevated tropospheric ozone concentration results in the decrease of bonds corresponding to glycerolipids (Kanter et al. 2013). This finding could have an implication in allergology, when we face unclear pathomechanism and allergy symptoms (Bashir et al. 2013).

Considering the obtained results, as well as the literature data, it can be concluded that the low air quality caused among others by vehicle pollutants influences the morphology and chemical composition of common mugwort pollen. The spectroscopic measurements allowed for identifying these chemical changes in pollen, which can lead to the increase of the risk sensitization in predisposed people and exacerbation of the symptoms in allergenic people (Sagai et al. 1996; de Weger et al. 2013; Ghiani et al. 2012). Our results confirmed that the FTIR and curve-fitting analysis of amide I profile could be successfully applied to assess the structural changes of common mugwort pollen collected from sites with different forms of anthropopression. FTIR is particularly sensitive to biological samples, and in this way, it can be considered as a tool for biomonitoring.

## Conclusions

The obtained results clearly show that air pollution, mainly caused by cars, could have an important impact not only on quantitative changes (different absorbance values of individual functional groups that build the chemical structures of pollen) but also qualitative changes (of the secondary structure of proteins) in common mugwort pollen. This finding was confirmed by the highest homology of the infrared spectra among individuals collected from the same site with distinct differences between sites with and without car traffic. The dendrogram shows it perfectly. Moreover, structural changes in proteins, observed in the second derivative of the FTIR spectrum and in the curve-fitting analysis of amide I profile, can be caused inter alia by air pollutants. This can lead to the formation of more aggressive allergenic proteins and hence to more frequent occurrence of allergy in people. A detailed investigation of pollen macromolecules may help in the allergy diagnosis, explaining the etiology of disease and then the immunotherapy.

Summarizing, FTIR spectroscopy is a sensitive indicator of chemical composition of pollen and can be helpful in biomonitoring, especially that pollution not always induces characteristic or evident symptoms in plants.
